# Surface-Functionalized Glass Nanoparticles with Algae-Derived Bio-Binder (ADBB) as Reinforcing Agent for Epoxy/ADBB Matrix Nanocomposite

**DOI:** 10.3390/polym17101334

**Published:** 2025-05-14

**Authors:** Abhijeet Mali, Torti Uwaike, Philip Agbo, Shobha Mantripragada, Lijun Wang, Lifeng Zhang

**Affiliations:** 1 Department of Nanoengineering, Joint School of Nanoscience and Nanoengineering, North Carolina A&T State University, 2907 E Gate City Blvd, Greensboro, NC 27401, USA; 2 Department of Natural Resources and Environmental Design, College of Agriculture and Environmental Sciences, North Carolina A&T State University, 1601 E Market St, Greensboro, NC 27411, USA

**Keywords:** glass nanoparticle, algae-derived bio-binder, surface-modifying agent, epoxy, sustainability

## Abstract

The algae-derived bio-binder (ADBB) from hydrothermal liquefaction has been reported to be an effective and sustainable new alternative to petroleum-based curing agents for epoxy resin. However, there is still room for the epoxy/ADBB system to attain the comprehensive mechanical performance of conventional epoxy-based nanocomposites, typically reinforced with surface-functionalized nanofillers (e.g., glass nanoparticles (GNPs)) by petroleum-based silane coupling agents. Herein, we explored the use of ADBB as an innovative surface-modifying agent to functionalize GNPs and evaluated the potential of ADBB surface-functionalized GNPs (ADBB-GNPs) as a reinforcing agent in the epoxy/ADBB matrix nanocomposite by comparing them to pristine GNPs and (3-aminopropyl) triethoxysilane (APTES) (a popular silane coupling agent) surface-modified GNPs (APTES-GNPs). The surface functionalization of GNPs with ADBB was carried out and characterized by scanning electron microscopy (SEM), dynamic light scattering (DLS), and Fourier-transform infrared spectroscopy (FTIR). Material performance including tensile, flexural, and Izod impact properties and thermal properties of the resulting epoxy/ADBB nanocomposites were investigated by corresponding ASTM mechanical test standards and thermogravimetric analysis (TGA). Our results revealed that the ADBB is a sustainable and effective surface-modifying agent that can functionalize GNPs. The obtained ADBB-GNPs significantly improved the mechanical performance of the epoxy/ADBB system at ultra-low loading (0.5 wt.%) by up to 42% and the maximum decomposition rate temperature increased from 419 °C to 422 °C, both of which outperformed APTES-GNPs. This research sheds light on developing sustainable surface-modifying agents for nanofillers to create high-performance sustainable polymer composite materials.

## 1. Introduction

Epoxy as a thermosetting polymer resin is widely used across various industries such as adhesives, automotive, aerospace, structural engineering, electronics, wind energy, and civil engineering [1,2]. However, epoxy resin alone typically lacks comprehensive mechanical and thermal performance for these demanding applications. To address these limitations, reinforcing agents are commonly incorporated into epoxy resin to enhance the mechanical and thermal properties of the resultant epoxy composites. With the development of nanotechnology, nano-scale fillers such as graphene, carbon nanotubes, electrospun nanofibers, titanium dioxide nanoparticles, etc., have been effectively incorporated into epoxy matrices with improved interfacial bonding and significant reinforcing effects [3,4,5,6]. Among these nanofillers, glass nanoparticles (GNPs) have been extensively utilized in epoxy matrix composites owing to their distinctive properties including lightweight nature, high strength and stiffness, high resistance to corrosion and chemicals, superior thermal stability, excellent electrical insulation, and cost-effectiveness [7,8]. However, GNPs tend to agglomerate at high loadings in epoxy matrix, leading to their poor dispersion in the epoxy matrix and the consequent mechanical performance degradation of the resultant composite materials [9]. To overcome this problem, surface functionalization is widely used for GNPs [10,11]. It is noteworthy that surface-modified GNPs with silane coupling agents have been employed in a wide range of applications such as construction material [12], biomedical use [13], heavy metal ion removal [14], coating [15], etc. In particular, surface-modified GNPs make epoxy-based nanocomposites even stronger and lighter due to enhanced interfacial bonding and the better stability of GNPs in the epoxy matrix [11,16].

Environmental concerns regarding petroleum-based epoxy resins, curing agents, and silane coupling agents include greenhouse gas emission, climate change, and limited petroleum resources [17]. As a result, there is an increasing demand for sustainable substitutes for petrochemical-based epoxy resin systems [18,19]. It is known that hydrothermal liquefaction (HTL) is a common processing technique to obtain bio-oil from biomass and is mainly used as an alternative source of energy (fuel) [20]. In our previous research, we acquired a bio-oil from green microalgae, i.e., *Chlorella vulgaris*, through HTL and used it for epoxy composite purposes with the term bio-binder [21]. We injected sustainability into the conventional epoxy resin system by replacing the petroleum-based epoxy curing agent with the algae-derived bio-binder (ADBB) containing amine (-NH_2_) functional groups. Compared to the conventional epoxy resin system, our epoxy/ADBB system with an ADBB loading of 35 wt.% of epoxy resin demonstrated superior mechanical performance and thermal stability. However, there is no report yet on further increasing the mechanical properties of the epoxy/ADBB system by using GNPs as a reinforcing nanofiller, as demonstrated by the numerous studies on conventional epoxy resin systems [22]. The ADBB possesses multiple functional groups such as -OH and -NH_2_ functional groups [21] that can interact with silanol (Si-OH) groups on the surface of GNPs and thus has the potential to act as a surface-modifying agent for GNPs.

For the first time, this research explores the surface functionalization of GNPs with the ADBB and examines the impact of ADBB surface-functionalized GNPs (ADBB-GNPs) on the mechanical performance of the epoxy/ADBB resin system when used as a reinforcing agent. The results were compared to the reinforcing effects of pristine GNPs and surface-modified GNPs with a common silane coupling agent APTES (APTES-GNPs) in the epoxy/ADBB system. This inaugural investigation provided insights into the surface modification of GNPs using sustainable resources and further uses of these GNPs as reinforcing agents for the development of novel sustainable polymer nanocomposites.

## 2. Materials and Methods

### 2.1. Materials

Green microalgae (*Chlorella vulgaris*) were purchased from nuts.com (Cranford, NJ, USA) for bio-binder synthesis. The epoxy resin (EPON 862) and its accompanying curing agent EPIKURE W were acquired from Miller Stephenson (Danbury, CT, USA). Glass nanoparticles (GNPs, i.e., SiO_2_ nanoparticles, with an average diameter of 20 nm, and BET surface area of 200 m^2^/g) and (3-aminopropyl) triethoxysilane (APTES, ≥98%) were bought from Millipore Sigma (St. Louis, MO, USA). Anhydrous acetone (≥99.5%) and ethanol (99.5%) were obtained from Fisher Scientific (Waltham, MA, USA). All chemicals were used as received.

### 2.2. Surface Functionalization of Glass Nanoparticles

The sustainable algae-derived bio-binder (ADBB) was prepared by the hydrothermal liquefaction of *Chlorella vulgaris*, as described in our previous report [21]. The ADBB was then employed as a surface modifier for GNPs. APTES, a common silane coupling agent, was also used to surface-modify GNPs for comparison purposes.

To surface-modify GNPs using the ADBB, the GNPs were first dispersed in ethanol at a weight fraction of 1.5 wt.%. The dispersion of GNPs in ethanol was then sonicated using a 500 W ultrasonic probe sonicator (QSONICA, Newtown, CT, USA) at 200 W power for 5 min to ensure a uniform dispersion. Further, a 3 wt.% ADBB in ethanol solution was added into the GNP dispersion and the system was stirred at 600 rpm for 10 min to prepare a homogeneous mixture. The pH of the solution was maintained at 4–5 by using acetic acid. To successfully graft ADBB molecules on GNPs, the surface modification reaction was carried out for 2 h with continuous stirring at 600 rpm. Finally, the mixture solution was centrifuged, and the precipitate was washed several times using ethanol to remove the physically absorbed ADBB and acquire the ADBB surface-functionalized GNPs (ADBB-GNPs). Using the same approach, GNPs were surface-modified with APTES and the obtained APTES surface-functionalized GNPs (APTES-GNPs) were used as a control sample. Prior to further use, all the surface-functionalized GNPs were dried in a vacuum oven at 60 °C for 8 h.

### 2.3. Fabrication of GNP-Reinforced Epoxy/ADBB Nanocomposite

The epoxy resin (EPON 862) and ADBB were used as the matrix phase and curing agent, respectively, at a ratio of 100/35 (epoxy/ADBB) with pristine and surface-modified GNPs as reinforcing agents to prepare GNP-reinforced epoxy/ADBB nanocomposites. The epoxy resin and ADBB were first heated and degassed separately at 60 °C for 10 min in a vacuumed oven. Then, GNPs were added into epoxy resin at respective loadings. The resultant mixture of the epoxy resin and GNPs was subjected to vigorous ultrasonication for 10 min using the ultrasonic probe sonicator at 200 W power for the homogeneous dispersion of GNPs in the epoxy resin. The ADBB was added into the epoxy resin afterwards at 35 wt.% of epoxy, and the mixture was stirred at 600 rpm and 60 °C for another 10 min to obtain a uniform mixture. The mixture of GNPs, the epoxy resin, and the ADBB was further sonicated for 5 min to ensure homogeneity. Subsequently, the obtained epoxy/ADBB/GNP system was degassed for 10 to 15 min at 60 °C under vacuum to remove air bubbles. After being degassed, the epoxy/ADBB/GNP system was poured into a mold and cured for 4 h at 400 °F [21]. For comparison, an epoxy/ADBB composite without GNPs was prepared using the same approach. Moreover, a conventional epoxy composite using EPON 862 epoxy and its paired curing agent EPIKURE W was prepared accordingly and cured based on the curing cycle recommended by the manufacturer as another control sample.

### 2.4. Characterization

A Zeiss (White Plains, NY, USA) Auriga Crossbeam FIB field emission scanning electron microscope (FESEM) was used to investigate the morphology of the GNPs and the fracture surfaces of the GNP-reinforced epoxy/ADBB nanocomposites. Prior to SEM, all the samples were sputter-coated with gold–palladium in a thickness of 7 nm to avoid charge accumulation. The average GNP diameter was determined by measuring the diameters of at least 30 randomly selected nanoparticles in SEM images using Image J software (version 1.53k). To confirm the surface modification of GNPs with the ADBB and APTES, FTIR was performed via an Agilent (Santa Clara, CA, USA) Varian 670 FTIR spectrometer over the wavenumber range of 4000–500 cm^−1^ with a total of 64 scans for each sample. The zeta potential and hydrodynamic diameter of both pristine and surface-functionalized GNPs were measured using dynamic light scattering (DLS) via the Malvern (Westborough, MA, USA) ZEN3600 Zetasizer. For the DLS measurement, GNPs were mixed with DI water at a weight ratio of 1:10,000. The GNP-DI water system was then sonicated for 5 min to achieve a homogeneous solution. DLS analysis was carried out with 1 mL of each as-prepared sample.

Mechanical properties of all epoxy/ADBB nanocomposites were investigated using tensile, flexural (three-point bending), and Izod impact tests following ASTM D638 Type-IV [23], ASTM D790 [24], and ASTM D256 [25] standards, respectively. Prior to mechanical tests, corresponding specimens were cut from the cured epoxy nanocomposite panels by a Flow (Kent, WA, USA) MACH 2 water jet cutting machine according to the respective ASTM standards. The tensile and flexural tests were performed on a UTM Instron (Norwood, MA, USA) 3384 universal testing machine and the Izod impact test was performed on an Impact 503 Tinius Olsen (Horsham, PA, USA) impact tester with five trials for each sample. An average value of each mechanical test result was reported with corresponding standard deviation.

The thermogravimetric analysis (TGA) of the epoxy/ADBB nanocomposites was conducted on a Q500 TGA (TA Instruments, New Castle, DE, USA) to investigate their thermal stability and decomposition temperatures. TGA was performed using a 10 mg sample from ambient temperature to 700 °C in a nitrogen atmosphere with a heating rate of 10 °C/min and a N_2_ flow rate of 50 mL/min.

## 3. Results and Discussion

### 3.1. Morphology of Pristine and Surface-Functionalized GNPs

**Figure 1:**
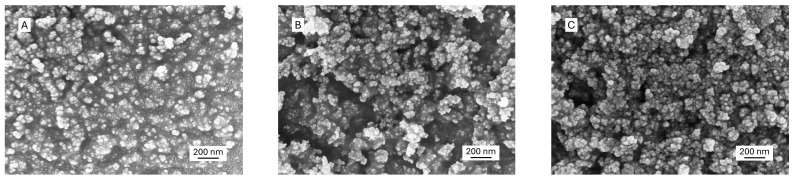
SEM images of pristine and surface-modified GNPs: (**A**) pristine GNPs; (**B**) APTES-GNPs; (**C**) ADBB-GNPs.

The morphology of pristine and surface-modified GNPs was examined by SEM (Figure 1). All the GNPs exhibited as agglomerates with an average size of 38 ± 11 nm, 41 ± 12 nm, and 43 ± 10 nm for pristine GNPs, APTES-GNPs, and ADBB-GNPs, respectively. Surface-modified GNPs with APTES and the ADBB showed more agglomeration, which was likely due to enhanced particle–particle interactions following the surface modification.

### 3.2. More Characterizations of ADBB-GNPs

**Figure 2:**
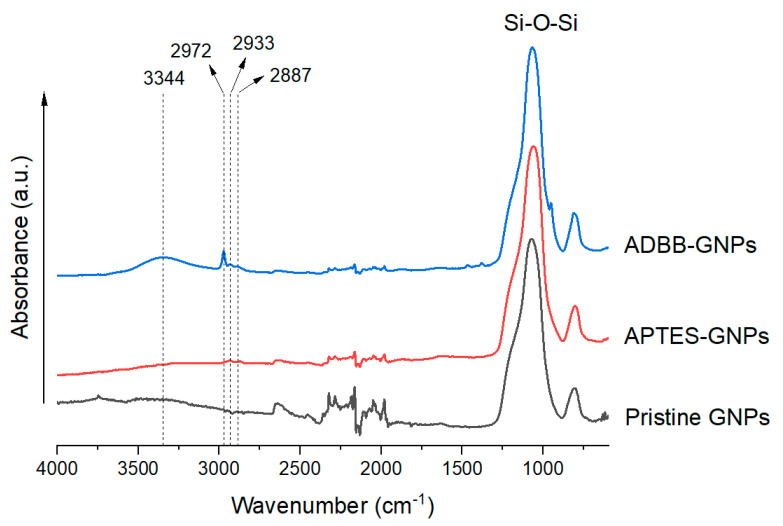
FTIR spectra of pristine GNPs, APTES-GNPs, and ADBB-GNPs.

FTIR was used to characterize the surface-functionalized GNPs to confirm the surface modification of GNPs with APTES and the ADBB (Figure 2). All the GNP samples showed a strong peak around 1070 cm^−1^, which can be attributed to the Si-O-Si bonds of GNPs. The FTIR spectrum of ADBB-GNPs exhibited a broad peak centered at 3344 cm^−1^, indicating the presence of N–H and O–H bonds, and peaks at 2972 cm^−1^, 2933 cm^−1^, and 2887 cm^−1^, indicating the presence of C–H bonds from –CH_3_, –CH_2_–, and –CH<. GNPs did not show these IR peaks, which confirmed the successful grafting of the ADBB to the SiO_2_ surface. The FTIR spectrum of APTES-GNPs also showed peaks in the range of 2800 cm^−1^–3000 cm^−1^, indicating the successful grafting of APTES to the SiO_2_ surface. It is noteworthy that ADBB grafting showed stronger peaks in this range, suggesting higher grafting efficiency. The grafting of the ADBB could be attributed to the chemical reactions between GNPs’ silanol functional groups (-Si-OH) and carboxylic acid (-COOH) and hydroxyl functional groups (-OH) from the ADBB [21].

**Figure 3:**
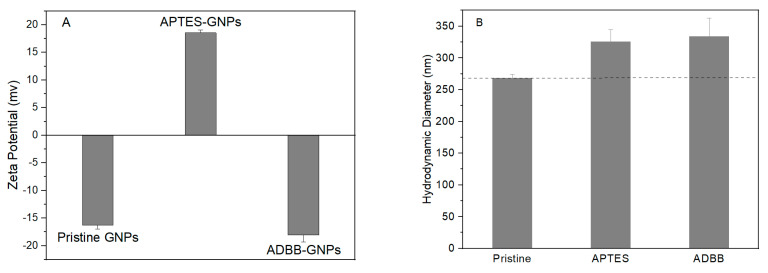
Zeta potential (**A**) and hydrodynamic diameter (**B**) of pristine GNPs, APTES-GNPs, and ADBB-GNPs using DLS.

The physical stability of surface-functionalized GNPs with APTES and the ADBB was investigated using DLS (Figure 3). Pristine GNPs showed an average zeta potential of −16 mV and an average hydrodynamic dimeter of 268 nm. ADBB-GNPs possessed slightly more negative zeta potential (−18 mV) and a larger hydrodynamic diameter (334 nm). For comparison, APTES-GNPs exhibited a similar hydrodynamic diameter of 326 nm but a positive zeta potential of +19 mV. ADBB-GNPs demonstrated similar physical stability to pristine GNPs and APTES-GNPs based on the absolute value of the zeta potential. This characteristic suggested a similar dispersion and stabilization of ADBB-GNPs in the epoxy matrix to previously studied pristine GNPs and APTES-GNPs [16].

### 3.3. Reinforcing Effect of Pristine GNPs in Epoxy/ADBB Nanocomposite

**Figure 4:**
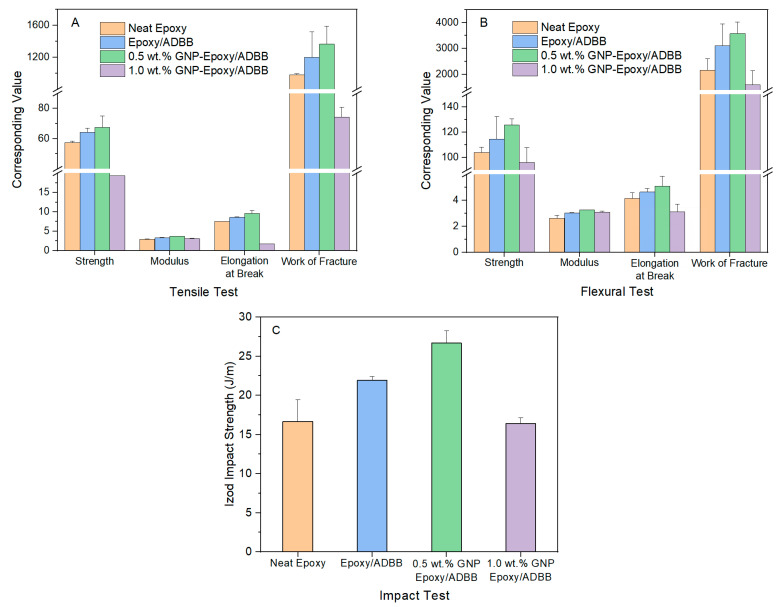
Comparison of tensile properties (**A**), flexural properties (**B**), and Izod impact strength (**C**) of neat epoxy, epoxy/ADBB, and pristine GNP-reinforced epoxy/ADBB nanocomposites at 0.5 wt.% and 1 wt.% loadings.

Based on our previous research findings regarding epoxy/ADBB formulation and GNP loading in GNP-reinforced epoxy nanocomposite [16,21], the mechanical properties, including tensile, flexural, and impact properties of the epoxy/ADBB (100/35, wt./wt.) nanocomposite reinforced by pristine GNPs at loading levels of 0.5 wt.% and 1.0 wt.% (labeled as 0.5 wt.% GNP-epoxy/ADBB and 1.0 wt.% GNP-epoxy/ADBB), were investigated to evaluate the reinforcing effect of pristine GNPs (Figure 4). The epoxy/ADBB composite without GNPs (labeled as epoxy/ADBB) was prepared and tested for comparison purposes. Moreover, the EPON 862 epoxy with its paired curing agent EPIKURE W (labeled as neat epoxy) was also prepared and tested as the second control sample.

The mechanical performance of the pristine GNP-reinforced epoxy/ADBB nanocomposites were evaluated first by a tensile test using tensile strength, Young’s modulus, elongation at break, and the work of fracture, respectively (Figure 4A). The incorporation of 0.5 wt.% GNPs significantly improved the load transfer capability, leading to approximately 5.1%, 9.9%, 12%, and 14% increments in tensile strength, Young’s modulus (stiffness), elongation at break (ductility), and the work of fracture (toughness), respectively, with respect to those of the epoxy/ADBB composite. Compared to neat epoxy, these enhancements were even more pronounced, i.e., ~17%, ~27%, ~28%, and ~40% improvements in tensile strength, Young’s modulus, elongation at break, and the work of fracture, respectively. However, an increase in GNP loading to 1 wt.% drastically deteriorated all the mechanical properties of the resultant epoxy/ADBB nanocomposite.

The response of pristine GNP-reinforced epoxy/ADBB nanocomposites to bending force was analyzed using a flexural test including flexural strength, modulus, elongation at break, and the work of fracture (Figure 4B). Specifically, the pristine GNP-reinforced epoxy/ADBB nanocomposite at 0.5 wt.% loading exhibited flexural strength at 126 MPa, flexural modulus at 3.3 GPa, flexural elongation at break at 5.1%, and a flexural work of fracture at 3576 kJ/m^3^, which were improvements of ~9.7%, ~6.9%, ~9.7%, and ~15%, respectively, compared to those of the epoxy/ADBB composite, as well as improvements of ~21%, ~24%, ~23%, and ~65%, respectively, with respect to those of the neat epoxy. Similarly, the 1 wt.% loading of pristine GNPs led to degraded flexural properties of the pristine GNP-reinforced epoxy/ADBB composite.

Compared to the impact absorption energy of neat epoxy and epoxy/ADBB composites, incorporating 0.5 wt.% pristine GNPs significantly increased the impact absorption energy of the resulting pristine GNP-reinforced epoxy/ADBB nanocomposite. The Izod impact strength increased by ~22% and ~61% with respect to that of epoxy/ADBB and neat epoxy, respectively (Figure 4C). This toughening effect of GNPs in epoxy resin could be ascribed to the debonding of GNPs and matrix shear banding [9]. More energy is needed to create micro-cracks in the process of fracture in the pristine GNP-reinforced epoxy/ADBB nanocomposite [26]. Unsurprisingly, the Izod impact strength of the pristine GNP-reinforced epoxy/ADBB nanocomposite at 1 wt.% GNP loading was even slightly less than that of the neat epoxy resin.

The mechanical property degradation of the pristine GNP-reinforced epoxy/ADBB nanocomposite at 1 wt.% GNP loading could be attributed to GNP agglomeration and the difficulty of homogeneous GNP distribution in the epoxy/ADBB system at this loading. It is well known that nanomaterials easily form agglomeration due to their ultrahigh specific area and consequently ultrahigh surface energy. Agglomeration naturally occurs for nanomaterials to reduce their surface energy. The agglomeration of nanoparticles in polymer particulate nanocomposites increases with nanoparticle content and nanoparticle size reduction [27]. Moreover, the epoxy/ADBB system with 1 wt.% GNP loading bore relatively greater viscosity before curing and generated difficulty for GNP distribution in the epoxy/ADBB matrix. Consequently, agglomerated and non-uniformly distributed GNPs in the cured nanocomposite could become defects and stress concentration sites, which could facilitate the start and propagation of cracks and promote premature failure, leading to poorer mechanical properties.

Overall, the pristine GNP-reinforced epoxy/ADBB nanocomposite at 0.5 wt.% GNP loading exhibited better mechanical properties than neat epoxy and epoxy/ADBB composites. The enhanced mechanical properties could be assigned to increased interfacial bonding between the epoxy/ADBB matrix and the well-distributed GNPs at this loading, caused by the ultrafine size and concomitantly ultrahigh specific surface area of the GNPs. GNPs could also act as barriers to crack propagation in the epoxy/ADBB matrix, boosting toughness and fracture resistance.

### 3.4. Reinforcing Effect of ADBB-GNPs in Epoxy/ADBB Nanocomposite

**Figure 5:**
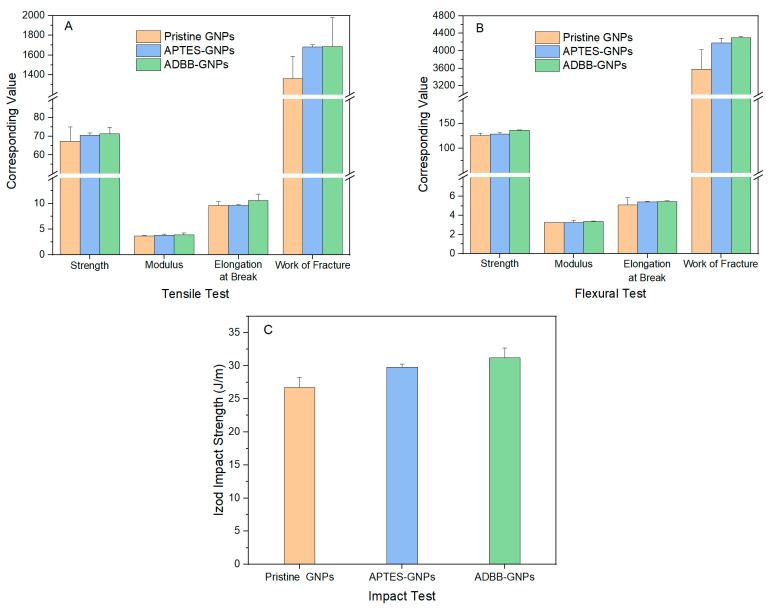
Comparison of tensile properties (**A**), flexural properties (**B**), and Izod impact strength (**C**) of pristine GNP- and surface-modified GNP-reinforced epoxy/ADBB nanocomposites at 0.5 wt.% loading.

Given the overall excellent mechanical performance of pristine GNP-reinforced epoxy/ADBB nanocomposite at 0.5 wt.% GNP loading, this loading was adopted to evaluate the reinforcing effect of ADBB-GNPs on the mechanical properties of the resultant epoxy/ADBB nanocomposite (Figure 5). The reinforcing effect of ADBB-GNPs was compared to that of APTES-GNPs to gain insights into using a sustainable ADBB as a novel surface-modifying agent versus petroleum-based silane coupling agents for glass fillers in developing polymer composites.

On top of the mechanical property improvement from the addition of pristine GNPs as shown in the previous section, the inclusion of surface-functionalized GNPs further improved the mechanical performance of the epoxy/ADBB nanocomposites. Compared to pristine GNPs, APTES-GNPs improved the tensile strength, Young’s modulus, elongation at break, and work of fracture of the resultant epoxy/ADBB nanocomposite in the tensile test by ~4.8%, ~3.4%, ~0.2%, and ~23%, respectively, while ADBB-GNPs improved these tensile mechanical properties by ~5.7%, ~6.7%, ~10%, and ~24% and reached ~71 MPa, ~3.9 GPa, ~11%, and ~1688 kJ/m^3^, respectively (Figure 5A). ADBB-GNPs generally outperformed APTES-GNPs in improving the tensile properties, especially ductility. Furthermore, ADBB-GNPs improved the flexural strength, modulus, elongation at break, and work of fracture of the resultant epoxy/ADBB nanocomposite by approximately 8.5%, 3.1%, 6.7%, and 20%, respectively, with respect to those of the pristine GNP-reinforced epoxy/ADBB nanocomposite, and reached approximately 136 MPa, 3.4 GPa, 5.4%, and 4300 kJ/m^3^, respectively (Figure 5B). For comparison, APTES-GNPs improved the corresponding flexural mechanical properties by approximately 2.4%, −0.4%, 6.4%, and 17%, respectively. Again, ADBB-GNPs outperformed APTES-GNPs in the flexural properties, especially strength and modulus. Additionally, ADBB-GNPs demonstrated an improvement in the Izod impact strength of the resultant epoxy/ADBB nanocomposite by ~17% with reference to pristine GNP-reinforced epoxy/ADBB nanocomposite and reached ~31 J/m, which exceeded the corresponding improvement (~12%) gained by using APTES-GNPs.

### 3.5. Reinforcing Mechanism

**Figure 6:**
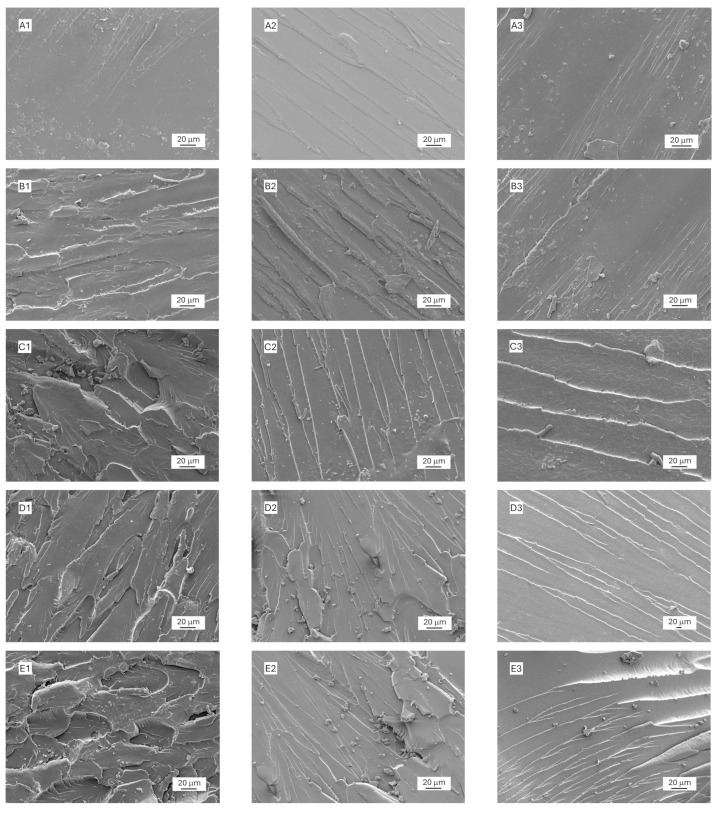
Fracture surface morphology of neat epoxy (**A**), epoxy/ADBB (**B**), pristine GNP-reinforced epoxy/ADBB (**C**), APTES-GNP-reinforced epoxy/ADBB (**D**), and ADBB-GNP-reinforced epoxy/ADBB (**E**) after mechanical tests. In total, 0.5 wt.% respective GNPs were loaded in (**C**–**E**). Label 1—tensile test; Label 2—flexural test; Label 3—Izod impact test.

To better understand the reinforcing effect of ADBB-GNPs in the epoxy/ADBB nanocomposite, the fracture surface of tensile, flexural, and Izod impact test specimens from neat epoxy, epoxy/ADBB, pristine GNP-reinforced epoxy/ADBB, APTES-GNP-reinforced epoxy/ADBB, and ADBB-GNP-reinforced epoxy/ADBB nanocomposites were examined by SEM (Figure 6).

It is apparent from Figure 6(A1–A3) that the neat epoxy samples showed a relatively smooth fracture surface with very few fracture lines from all mechanical tests, indicating low toughness. Epoxy/ADBB without GNPs exhibited a much rougher surface after mechanical tests (Figure 6(B1–B3)), indicating improved toughness. The incorporation of GNPs into the epoxy/ADBB matrix further improved its toughness, as indicated by its even rougher surface after mechanical tests (Figure 6C–E). ADBB-GNPs (Figure 6(E1–E3)) provided superior fracture toughness when used as a reinforcing agent in the epoxy/ADBB nanocomposite as evidenced by the more pronounced rough fracture surface features that outperformed pristine GNPs (Figure 6(C1–C3)) and even APTES-GNPs (Figure 6(D1–D3)), highlighting the effectiveness of the ADBB in surface-functionalizing GNPs for reinforcing applications.

Overall, the sustainable ADBB surpassed the commercial silane coupling agent APTES when used as a surface-modifying agent for GNPs to reinforce the epoxy/ADBB matrix. The fundamental reasons can be attributed to its better compatibility and more possible chemical reactions between ADBB-GNPs and the epoxy/ADBB matrix. The ADBB is a complicated mixture of amines, alcohols, amides, esters, fatty acids, aromatic rings, etc. [21]. The ADBB molecules on the GNP surface have more affinity with the matrix due to their similar chemical structure to both epoxy (e.g., aromatic rings) and ADBB molecules in the matrix. Furthermore, due to their multifunctional feature, there are more chances for the ADBB molecules on the GNP surface to have chemical reactions with epoxide functional groups in the epoxy matrix through amine (-NH_2_), carboxylic acid (-COOH), or hydroxyl (-OH) functional groups and with ADBB molecules in the matrix (the epoxy/ADBB system contained a significant amount of the ADBB, i.e., 35 wt.% of epoxy). As a result, interfacial bonding between the ADBB-GNP nanofiller and epoxy/ADBB matrix was significantly enhanced, which facilitated better stress transfer. In addition, these reactions between ADBB-GNPs and the epoxy/ADBB matrix could effectively prevent the agglomeration of ADBB-GNPs and lead to the better stability of ADBB-GNPs in the matrix. The better-dispersed ADBB-GNPs in the epoxy/ADBB matrix could delay crack initiation, obstruct crack propagation, and force cracks to take a more tortuous path, all of which increase the energy required for crack propagation. Furthermore, the strong interfacial bonding between the ADBB-GNP filler and the epoxy/ADBB matrix allowed for efficient energy dissipation through debonding, pull-out, and plastic deformation. All these factors together resulted in the higher toughness of the ADBB-GNP-reinforced epoxy/ADBB nanocomposite.

For comparison, APTES-GNPs only have -NH_2_ functional groups and lack aromatic functional groups, which thus work not as efficiently as ADBB-GNPs from the point of view of interfacial bonding in the nanocomposite.

### 3.6. Thermal Properties of ADBB-GNP-Reinforced Epoxy/ADBB Nanocomposite

**Figure 7:**
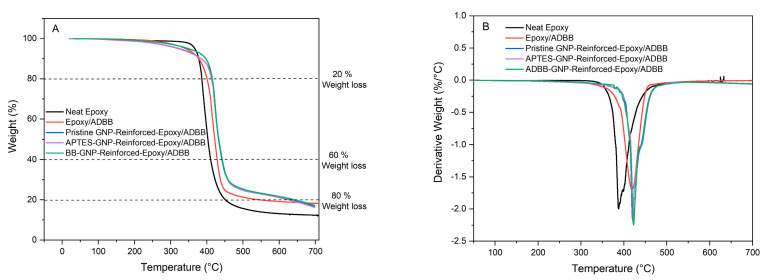
Weight loss (**A**) and derivative weight loss (**B**) vs. temperature of neat epoxy, epoxy/ADBB, pristine GNP-reinforced epoxy/ADBB, APTES-GNP-reinforced epoxy/ADBB, and ADBB-GNP-reinforced epoxy/ADBB nanocomposites from TGA.

Thermogravimetric analysis (TGA) was performed to determine the decomposition temperature and thermal stability of the ADBB-GNP-reinforced epoxy/ADBB nanocomposite. For a thorough comparison, the TGA results of neat epoxy, epoxy/ADBB, pristine GNP-reinforced epoxy/ADBB, APTES-GNP-reinforced epoxy/ADBB, and ADBB-GNP-reinforced epoxy/ADBB at 0.5 wt.% GNP loading are included in Figure 7.

Compared to neat epoxy (i.e., EPON 862 with curing agent EPIKURE W), using the ADBB instead of EPICURE W showed a similar temperature at 10% weight loss but resulted in significantly improved thermal stability at a higher temperature, i.e., the temperatures at 20%, 60%, and 80% weight loss increased from 386 °C to 400 °C, 410 °C to 428 °C, and 451 °C to 545 °C, respectively. The inclusion of pristine GNPs or APTES-GNPs in the epoxy/ADBB nanocomposite further increased its thermal stability and exhibited similar improvement. ADBB-GNPs outperformed pristine GNPs and APTES-GNPs in thermal stability improvement in the resultant epoxy/ADBB nanocomposite. Specifically, compared to APTES-GNP-reinforced epoxy/ADBB, the temperatures at 10%, 20%, 60%, and 80% weight loss increased from 384 °C to 398 °C, 413 °C to 415 °C, 442 °C to 443 °C, and 626 °C to 643 °C for the ADBB-GNP-reinforced epoxy/ADBB composite. On top of the TGA weight loss results as described above (Figure 7A), the derivative thermogravimetric (DTG) analysis (Figure 7B) showed a similar trend. For the neat epoxy, the maximum decomposition rate temperature (DTG peak) was observed at 388 °C. This temperature increased to 419 °C for epoxy/ADBB and further increased to 420 °C for pristine GNP-reinforced epoxy/ADBB and to 421 °C for APTES-GNP-reinforced epoxy/ADBB. The ADBB-GNP-reinforced epoxy/ADBB showed the highest maximum decomposition rate temperature (422 °C).

Compared to epoxy/ADBB, the enhanced thermal stability of the ADBB-GNP-reinforced epoxy/ADBB nanocomposite could be due to the role of ADBB-GNPs as a thermal barrier. GNPs (SiO_2_) have a high melting point (>1600 °C), while the aromatic components in the ADBB also have good thermal stability, both of which could act as a barrier to the thermal decomposition of epoxy, delay the epoxy’s degradation process, and slow down the rate of weight loss of the epoxy/ADBB nanocomposite upon heating. It is clear that ADBB-GNPs outperformed APTES-GNPs in improving the epoxy/ADBB nanocomposite’s thermal stability. This could be ascribed to the fact that the ADBB contains aromatic components that are more thermally stable than APTES molecules.

## 4. Conclusions

Glass nanoparticles (GNPs) were successfully surface-modified with the algae-derived bio-binder (ADBB) that was derived from green microalgae through hydrothermal liquefaction (HTL). The ADBB surface-functionalized GNPs (ADBB-GNPs) demonstrated great potential as an innovative reinforcing nanofiller in the new epoxy/ADBB matrix composite materials and outperformed both pristine GNPs and the GNPs modified by a popular silane coupling agent, APTES (APTES-GNPs).

Compared to epoxy/ADBB, the inclusion of pristine GNPs at 0.5 wt.% loading in epoxy/ADBB improved tensile strength, modulus, elongation at break, and the work of fracture by ~5.1%, ~9.9%, ~12%, and ~14%, respectively, and flexural strength, modulus, elongation at break, and the work of fracture by ~9.7%, ~6.9%, ~9.7%, ~15%, respectively, and it increased the Izod impact strength by ~22%. At the same loading, APTES-GNPs outperformed pristine GNPs as expected from the point of view of the reinforcing effect, and ADBB-GNPs further exceeded APTES-GNPs in this aspect. Specifically, the ADBB-GNP-reinforced epoxy/ADBB nanocomposites demonstrated superior tensile properties and possessed a tensile strength of ~71 MPa, Young’s modulus of ~3.9 GPa, elongation at break of ~11%, and work of fracture of ~1688 kJ/m^3^, which were improvements of ~11%, ~17%, ~23%, and ~41%, respectively, with respect to those of the epoxy/ADBB. Furthermore, ADBB-GNPs improved the flexure properties of the resultant epoxy/ADBB nanocomposite including flexural strength, modulus, elongation at break, and the work of fracture by approximately ~19%, ~10%, ~17%, and ~38%, respectively, with respect to those of epoxy/ADBB, and reached approximately 136 MPa, 3.4 GPa, 5.4%, and 4300 kJ/m^3^, respectively. In addition, the ADBB-GNP-reinforced epoxy/ADBB nanocomposite demonstrated an improvement of ~42% with respect to epoxy/ADBB regarding the Izod impact strength and reached ~31 J/m. The enhanced mechanical properties from ADBB-GNP-reinforced epoxy/ADBB and its outperformance over APTES-GNP-reinforced epoxy/ADBB could be attributed to stronger interfacial bonding between ADBB-GNPs and the epoxy/ADBB matrix and the better stability of ADBB-GNPs in the epoxy/ADBB matrix caused by better compatibility and more possible linking reactions between the ADBB and epoxy/ADBB.

It is noteworthy that the ADBB exceeded APTES, a popular commercially available silane coupling agent, in GNP surface modification for overall mechanical reinforcement in the resultant epoxy/ADBB nanocomposite, and it is an environmentally friendly and sustainable alternative to petroleum-based coupling agents for the surface modification of glass fillers in composite uses. Its renewable nature, cost-effectiveness, and surface reaction capability to composite fillers underscore the potential of the ADBB in developing high-performance environmentally friendly polymer composite materials.

## Data Availability

The original contributions presented in this study are included in the article. Further inquiries can be directed to the corresponding authors.
